# Supplementary material for: Hop (Humulus lupulus L.) Phenolic Compounds Profile Depends on Cultivar and Plant Organ Maturity
Source: Molecules. 2025 May 29;30(11):2365. doi: 10.3390/molecules30112365 (PMC12155585; doi:10.3390/molecules30112365)
Supplement: Supplementary file 1 [file molecules-30-02365-s001.zip › molecules-3632326-supplementary.pdf]

Hop (*Humulus lupulus* L.) Phenolics Profile Depend on Cultivar and Anatomical Plant Organ Maturity

Jakub Piekara [1,*], Dorota Piasecka-Kwiatkowska [1], Hanna Hołaj [2], Małgorzata Jędryczka [3] and Oluwafemi Daniel Daramola [1] and Krzysztof Dwiecki [1,*]

[1] Department of Food Biochemistry and Analysis, Poznan University of Life Sciences, Mazowiecka 48, 60-623 Poznań, Poland; dorota.piasecka-kwiatkowska@up.poznan.pl (D.P.-K.); 89295@student.up.poznan.pl (O.D.D.)
[2] Agricultural Experimental Station 'Jastków', Panieńszczyzna, Chmielowa 5, 21-002 Jastków, Poland; rzd.jastkow@iung.pulawy.pl
[3] Institute of Plant Genetics, Polish Academy of Sciences, Strzeszyńska 34, 60-479 Poznań, Poland; mjed@igr.poznan.pl
* Correspondence: jakub.piekara@up.poznan.pl (J.P.); krzysztof.dwiecki@up.poznan.pl (K.D.); Tel.: +48-61-848-73-59 (J.P.); +48-61-848-73-65 (K.D.)

1. Comparison of the content of phenolic compounds between levels in each analysed hop cultivars

Table S1. Comparison of the content of phenolic compounds in cones in relation to level within the Marynka.

[mg / 100 g]	Lower	Middle	Upper
Gallic acid	7.03 ± 1.60 a	7.77 ± 1.01 a	7.55 ± 0.64 a
p-coumaric acid	6.79 ± 0.55 a	6.23 ± 0.81 a	7.36 ± 2.49 a
Ferulic acid	5.44 ± 0.12 a	6.45 ± 0.42 b	5.25 ± 0.17 a
Caffeic acid	29.03± 3.80 a	31.13 ± 5.19 a	40.01 ± 8.28 a
Protocatechuic acid	6.79 ± 0.55 a	6.23 ± 0.81 a	7.36 ± 2.49 a
Chlorogenic acid	23.23 ± 0.76 a	28.80 ± 0.32 a	27.43 ± 4.56 a
Sinapic acid	8.06 ± 0.78 a	5.14 ± 1.16 a	5.71 ± 2.00 a
Rutin	184.18 ± 5.19 a	209.50 ± 5.75 b	197.62 ± 11.71 ab
Kaempferol-3-glu	78.46 ± 1.58 a	103.14 ± 2.94 c	86.30 ± 2.28 b
Phenolic acid - total	83.27 ± 9.54 a	89.57 ± 9.52 a	97.27 ± 7.36 a
Flavonoids - total	274.31 ± 7.07 a	331.78 ± 7,95 b	313.55 ± 43.94 ab
Phenolic compounds – total	357,59 ± 13.48 b	421.35 ± 18.07 a	410.82 ± 40.10 a

Table S2. Comparison of the content of phenolic compounds in leaves in relation to level within the Marynka.

[mg / 100 g]	Lower	Middle	Upper
Gallic acid	19.45 ± 0.32 c	12.50 ± 1.95 a	15.99 ± 0.98 b
p-coumaric acid	4.12 ± 0.43 a	1.77 ± 0.46 b	3.80 ± 0.02 a
Ferulic acid	6.47 ± 0.16 a	8.18 ± 0.49 b	12.59 ± 0.21 c
Caffeic acid	19.42 ± 1.42 a	16.22 ± 0.38 a	39.08 ± 3.55 b
Protocatechuic acid	10.11 ± 0.16 c	6.50 ± 1.01 a	8.31 ± 0.51 b
Chlorogenic acid	2.43 ± 0.41 a	15.63 ± 6.53 b	45.09 ± 1.89 c
Sinapic acid	4.35 ± 0.32 b	3.98 ± 0.38 ab	3.31 ± 0.30 a
Rutin	62.65 ± 0.51 a	139.09 ± 8.20 b	241.78 ± 12.24 c
Kaempferol-3-glu	91.21 ± 3.46 a	126.90 ± 7.61 a	296.78 ± 18.48 b
Phenolic acid - total	68.51 ± 2.19 a	67.97 ± 7.41 a	165.25 ± 10.08 b
Flavonoids - total	178.43 ± 2.11 a	314.02 ± 9.42 b	586.32 ± 17.63 c
Phenolic compounds – total	246.94 ± 2.94 a	378.81 ± 13.35 a	751.57 ± 23.08 b

Table S3. Comparison of the content of phenolic compounds in stalks in relation to level within the Marynka.

[mg / 100 g]	Lower	Middle	Upper
Gallic acid	2.48 ± 0.21 a	2.89 ± 0.33 a	3.93 ± 0.75 a
p-coumaric acid	1.48 ± 0.47 a	1.91 ± 0.28 a	2.29 ± 0.18 a
Ferulic acid	3.18 ± 1.19 b	3.54 ± 0.18 c	5.26 ± 0.16 c
Caffeic acid	9.16 ± 1.22 a	16.05 ± 3.24 a	14.74 ± 4.30 a
Protocatechuic acid	2.51 ± 0.22 b	3.26 ± 0.19 a	3.52 ± 0.34 a
Gentisic acid	12.07 ± 1.16 a	10.31 ± 0.07 c	14.49 ± 0.23 b
Chlorogenic acid	6.29 ± 1.15 a	11.63 ± 2.04 a	9.01 ± 3.42 ab
Rutin	16.12 ± 1.33 a	15.11 ± 1.83 a	29.23 ± 8.05 ab
Kaempferol-3-glu	8.68 ± 0.11 a	7.75 ± 0.22 a	16.15 ± 4.84 a
Phenolic acid - total	37.17 ± 3.91 b	46.33 ± 3.22 a	53.24 ± 0.67 a
Flavonoids - total	24.81 ± 1.37 a	22.86 ± 2.03 a	45.38 ± 12.89 ab
Phenolic compounds – total	61.98 ± 5.54 a	72.45 ± 4.60 a	98.62 ± 12.30 b

Table S4. Comparison of the content of phenolic compounds in cones in relation to level within the Lubelski.

[mg / 100 g]	Lower	Middle	Upper
Gallic acid	6.10 ± 1.24 a	10.74 ± 0.47 b	4.79 ± 0.68 a
p-coumaric acid	4.77 ± 0.69 a	7.53 ± 1.09 b	6.38 ± 0.74 ab
Ferulic acid	7.14 ± 3.26 a	13.31 ± 4.84 a	11.08 ± a
Caffeic acid	15.60 ± 5.09 a	42.91 ± 1.61 c	29.64 ± 3.62 b
Protocatechuic acid	3.17 ± 0.64 a	5.58 ± 0.24 b	2.49 ± 0.35 a
Chlorogenic acid	9.24 ± 3.72 b	18.89 ± 0.21 a	18.12 ± 0.57 a
Sinapic acid	14.16 ± 1.28 b	11.79 ± 1.90 ab	9.69 ± 0.91 a
Rutin	78.62 ± 2.32 a	121.63 ± 1.21 a	123.52 ± 0.66 a
Kaempferol-3-glu	57.69 ± 0.45 a	72.36 ± 0.10 a	74.48 ± 0.46 a
Phenolic acid - total	60.20 ± 9.36 a	110.79 ± 8.19 a	82.22 ± 7.71 a
Flavonoids - total	211.70 ± 31.19 a	253.49 ± 5.08 a	285.03 ± 4.11 a
Phenolic compounds – total	271.90 ± 54.31 a	364.29 ± 6.63 a	367.25 ± 35.77 a

Table S5. Comparison of the content of phenolic compounds in leaves in relation to level within the Lubelski.

[mg / 100 g]	Lower	Middle	Upper
Gallic acid	7.53 ± 2.39 b	17.85 ± 0.78 a	14.23 ± 0.71 a
p-coumaric acid	0.87 ± 0.22 a	2.56 ± 0.62 b	4.11 ± 0.77 c
Ferulic acid	7.13 ± 0.85 a	12.11 ± 1.03 b	8.40 ± 1.47 a
Caffeic acid	14.30 ± 2.53 b	23.83 ± 3.04 a	30.91 ± 3.45 a
Protocatechuic acid	3.98 ± 1.24 b	9.28 ± 0.40 a	7.40 ± 0.36 a
Chlorogenic acid	14.30 ± 2.53 b	23.83 ± 3.04 a	30.91 ± 3.45 a
Sinapic acid	3.60 ± 0.78 a	5.12 ± 1.56 a	4.43 ± 1.57 a
Rutin	19.80 ± 2.67 a	74.93 ± 7.05 b	99.57 ± 13.43 c
Kaempferol-3-glu	75.66 ± 9.37 b	219.63 ± 15.59 a	244.09 ± 21.48 a
Phenolic acid - total	47.36 ± 1.99 b	106.10 ± 2.69 a	107.44 ± 6.44 a
Flavonoids - total	120.27 ± 17.35 a	412.66 ± 43.47 b	523.73 ± 37.24 c
Phenolic compounds – total	167.63 ± 15.12 a	518.77 ± 42.95 b	631.17 ± 44.46 c

Table S6. Comparison of the content of phenolic compounds in stalks in relation to level within the Lubelski.

[mg / 100 g]	Lower	Middle	Upper
Gallic acid	4.61 ± 0.20 a	9.23 ± 0.80 b	5.64 ± 1.30 a
p-coumaric acid	1.73 ± 0.15 a	2.49 ± 0.09 b	1.87 ± 0.07 a
Ferulic acid	1.12 ± 0.20 a	1.52 ± 0.03 b	2.43 ± 0.08 b

Caffeic acid	14.57 ± 2.19 b	19.54 ± 0.95 b	17.84 ± 1.22 a
Protocatechuic acid	2.74 ± 0.67 a	4.21 ± 0.24 b	2.98 ± 0.18 a
Gentisic acid	4.27 ± 0.36 b	3.60 ± 0.25 a	7.38 ± 3.35 a
Chlorogenic acid	6.83 ± 0.61 a	9.02 ± 1.30 a	7.21 ± 0.66 a
Rutin	26.10 ± 1.66 b	28.31 ± 2.51 c	40.93 ± 0.42 b
Kaempferol-3-glu	7.64 ± 0.79 a	8.35 ± 0.92 a	9.08 ± 3.28 a
Phenolic acid - total	35.87 ± 0.06 b	40.58 ± 1.73 a	44.98 ± 0.88 a
Flavonoids - total	40.11 ± 2.67 b	41.28 ± 6.28 b	60.62 ± 8.73 b
Phenolic compounds – total	78.98 ± 4.47 a	81.86 ± 7.72 ab	105.60 ± 9.48 b

Table S7. Comparison of the content of phenolic compounds in cones in relation to level within the Magnum.

[mg / 100 g]	Lower	Middle	Upper
Gallic acid	2.38 ± 0.20 a	2.07 ± 0.62 a	1.58 ± 0.24 b
p-coumaric acid	2.32 ± 0.35 a	3.25 ± 0.36 a	5.11 ± 0.40 b
Ferulic acid	0.84 ± 0.18 a	7.15 ± 1.40 b	1.60 ± 0.11 a
Caffeic acid	4.23 ± 0.48 a	9.11 ± 2.38 b	22.90 ± 0.50 c
Protocatechuic acid	1.23 ± 0.11 a	1.07 ± 0.32 a	0.82 ± 0.12 a
Chlorogenic acid	2.19± 0.41 a	5.19 ± 0.27 b	11.38 ± 0.44 c
Sinapic acid	8.63 ± 1.13 a	10.90 ± 1.05 a	8.25 ± 1.47 a
Rutin	15.68 ± 1.39 a	18.89 ± 0.51 a	43.17 ± 2.18 b
Kaempferol-3-glu	7.22 ± 0.45 b	23.66 ± 1.31 a	36.92 ± 6.30 a
Phenolic acid - total	30.68 ± 0.92 a	47.46 ± 6.67 b	63.15 ± 1.72 c
Flavonoids - total	31.00 ± 0.45 a	55.30 ± 3.39 b	87.23 ± 14.31 c
Phenolic compounds – total	61.68 ± 15.71 a	102.76 ± 9.79 b	150.39 ± 13.57 c

Table S8. Comparison of the content of phenolic compounds in leaves in relation to level within the Magnum.

[mg / 100 g]	Lower	Middle	Upper
Gallic acid	8.58 ± 2.21 a	11.17 ± 1.01 a	18.41 ± 4.01 b
p-coumaric acid	1.15 ± 0.38 a	4.40 ± 0.07 a	16.69 ± 3.37 b
Ferulic acid	3.85 ± 0.05 b	15.56 ± 1.42 a	12.45 ± 2.86 a
Caffeic acid	7.58 ± 3.43 a	24.73 ± 1.26 a	96.26 ± 17.02 b
Protocatechuic acid	4.46 ± 1.15 a	5.81 ± 0.52 a	9.58 ± 2.08 b
Chlorogenic acid	5.68 ± 2.61 a	19.89 ± 2.34 b	59.15 ± 1.30 c
Sinapic acid	3.43 ± 0.72 a	4.59 ± 1.72 a	3.34 ± 1.22 a
Rutin	27.07 ± 0.01 a	133.17± 14.89 b	293.32 ± 14.61 c
Kaempferol-3-glu	50.04 ± 0.31 a	191.33 ± 15.58 b	328.62 ± 5.35 c
Phenolic acid - total	34.75 ± 7.55 a	86.15 ± 3.72 b	215.90 ± 27.07 c
Flavonoids - total	89.93 ± 0.13 a	357.17 ± 9.37 b	706.12 ± 15.64 c
Phenolic compounds – total	124.68 ± 2.31 a	443.33 ± 11.87 b	922.03 ± 43.29 c

Table S9. Comparison of the content of phenolic compounds in stalks in relation to level within the Magnum.

[mg / 100 g]	Lower	Middle	Upper
Gallic acid	16.28 ± 2.44 b	16.23 ± 1.32 c	25.97 ± 7.29 b
p-coumaric acid	1.31 ± 0.11 a	1.97 ± 0.26 ab	4.24 ± 0.46 b
Ferulic acid	1.39 ± 0.30 ab	0.73 ± 0.12 a	1.43 ± 0.11 a
Caffeic acid	7.50 ± 0.32 a	12.65 ± 0.49 a	13.53 ± 0.51 a

Protocatechuic acid	4.00 ± 0.32 a	4.64 ± 0.27 a	3.61 ± 0.94 a
Gentisic acid	10.48 ± 0.50 a	6.12 ± 0.34 b	5.92 ± 0.10 a
Chlorogenic acid	6.33 ± 1.42 a	10.52 ± 2.67 a	14.14 ± 2.72 c
Rutin	17.77 ± 0.67 a	22.81 ± 1.84 b	27.13 ± 1.84 a
Kaempferol-3-glu	6.75 ± 1.21 a	3.72 ± 1.14 b	9.01 ± 1.42 a
Phenolic acid - total	47.29 ± 1.64 a	52.91 ± 2.70 a	68.84 ± 10.44 b
Flavonoids - total	22.18 ± 2.01 a	27.38 ± 3.14 a	36.14 ± 4.26 a
Phenolic compounds – total	68.47 ± 3.63 a	80.29 ± 2.18 a	104.98 ± 14.31 b

2. Spectra of not identified flavonoids

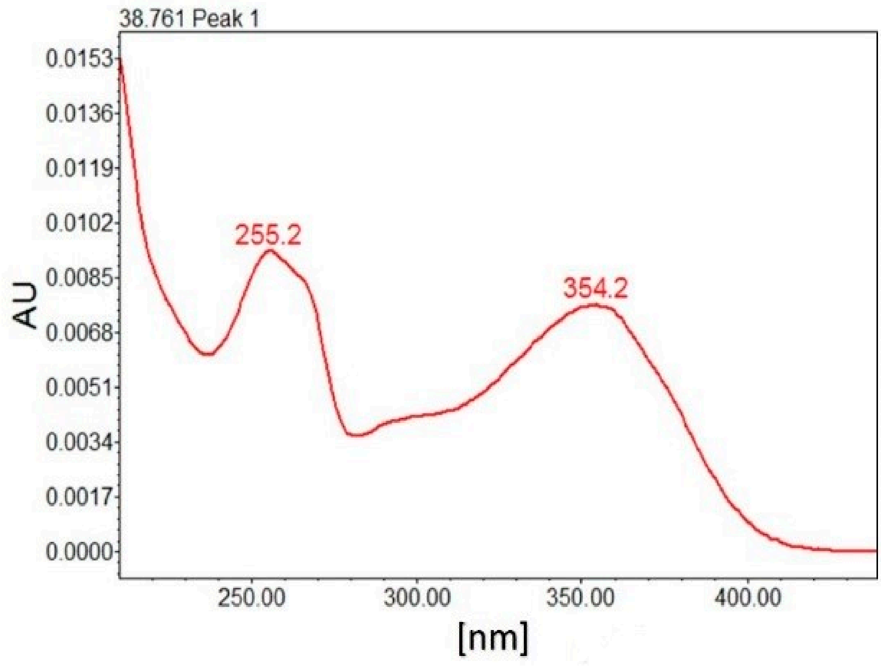

Figure S1. UV-Vis spectrum of not identified flavonoid 1.

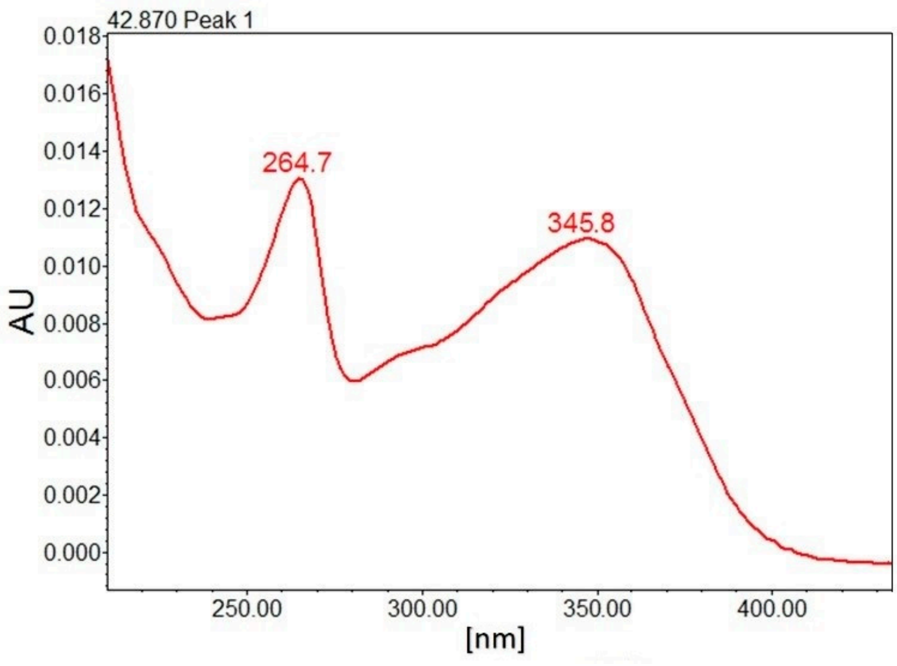

Figure S2. UV-Vis spectrum of not identified flavonoid 2.

3. Chromatograms of phenolic compounds standards

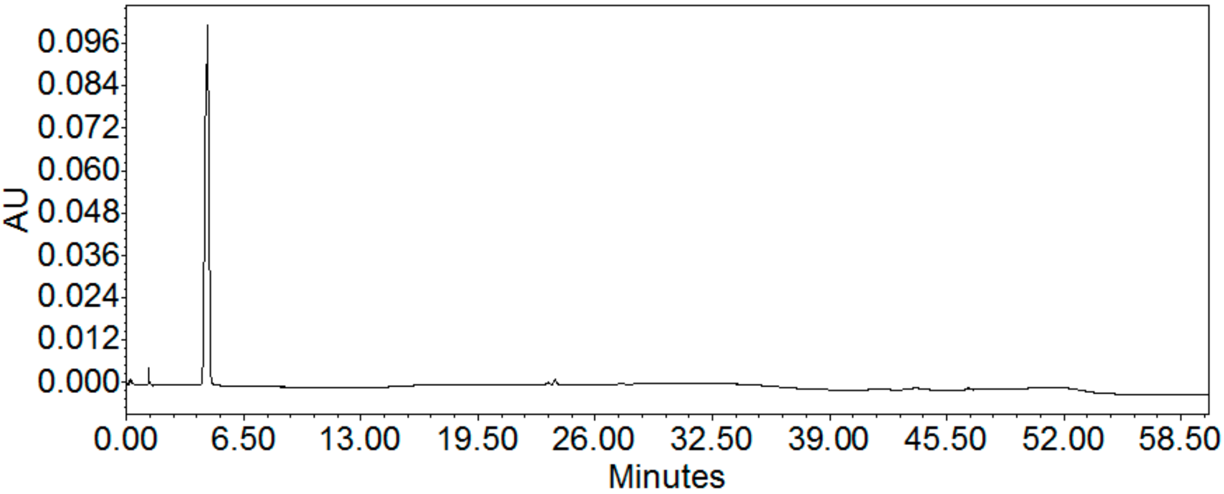

Figure S3. Chromatogram of gallic acid standard

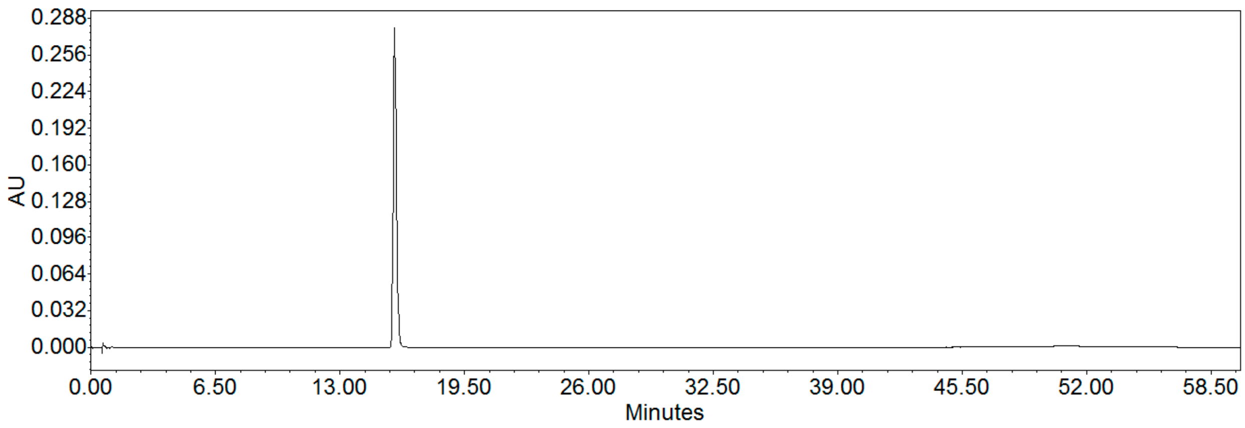

Figure S4. Chromatogram of p-coumaric standard

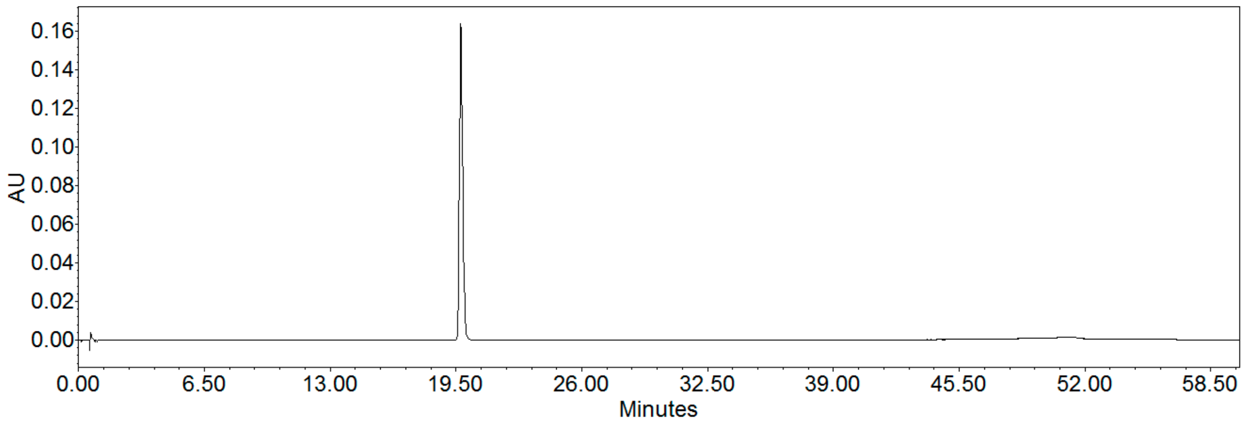

Figure S5. Chromatogram of ferulic acid standard

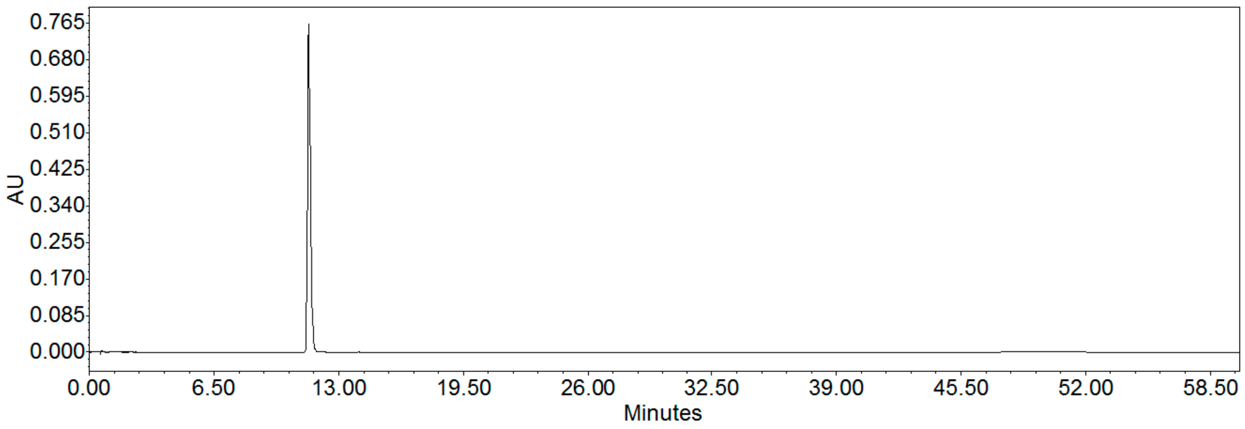

Figure S6. Chromatogram of caffeic acid standard

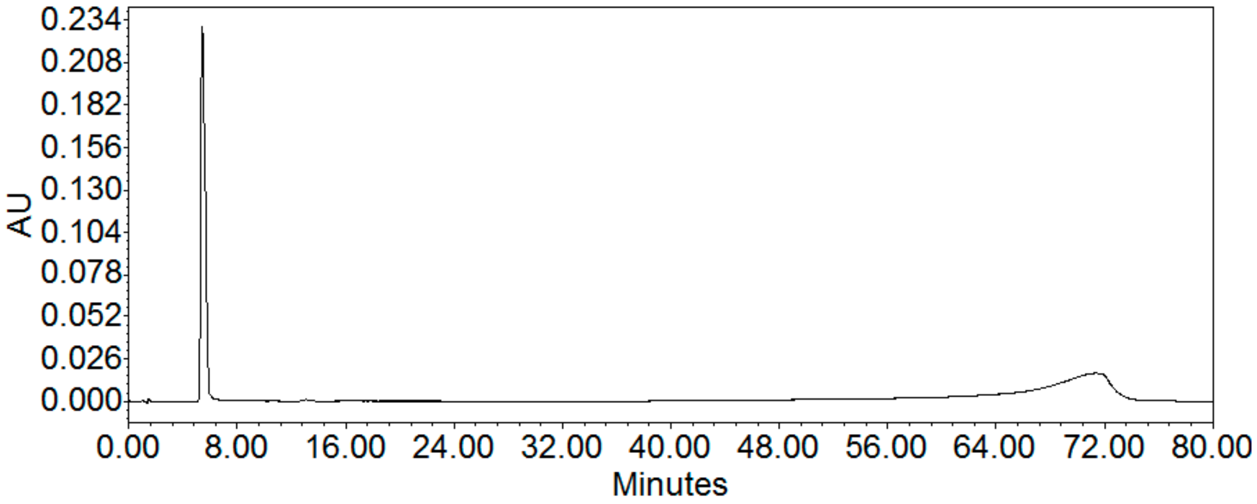

Figure S7. Chromatogram of protocatechuic standard

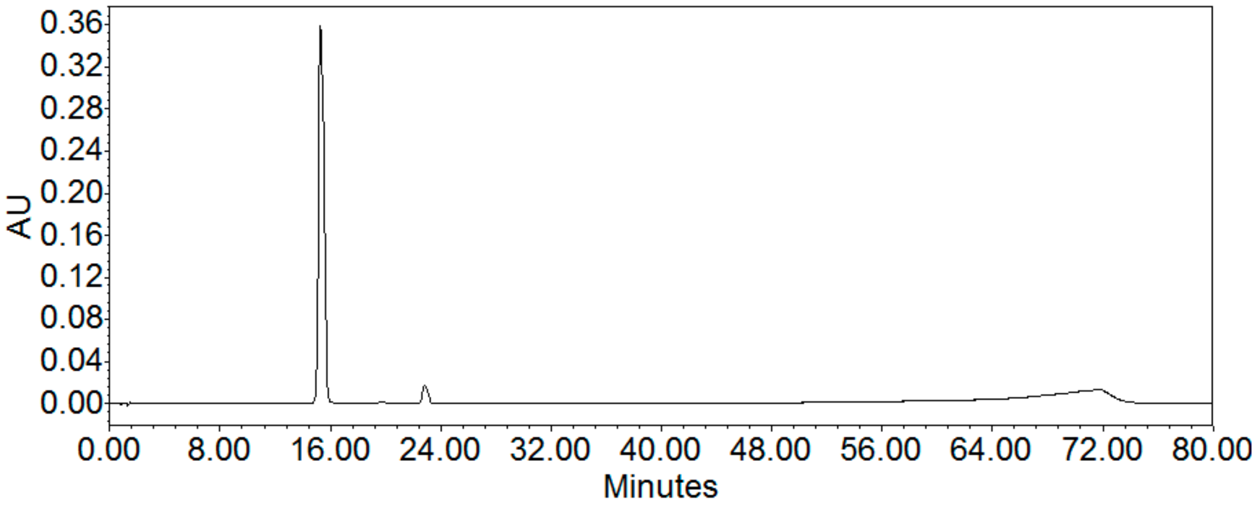

Figure S8. Chromatogram of chlorogenic acid standard

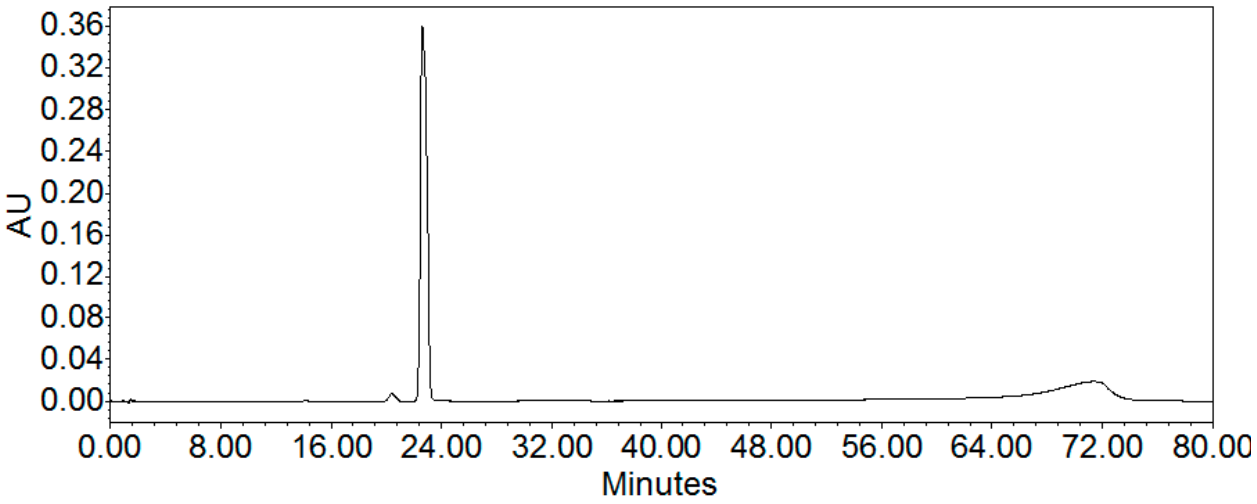

Figure S9. Chromatogram of sinapic acid standard

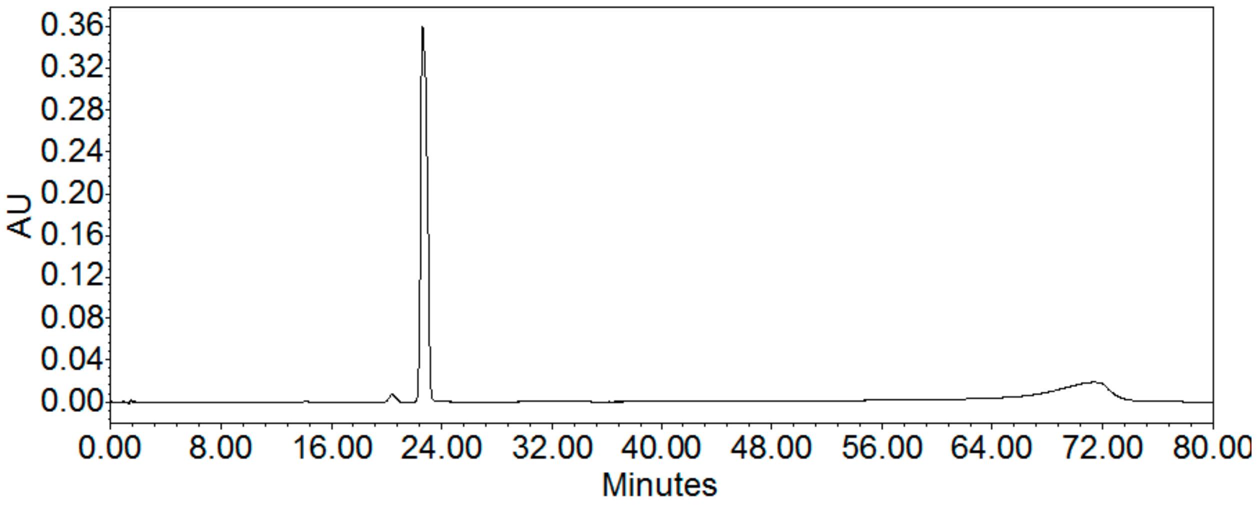

Figure S10. Chromatogram of rutin standard

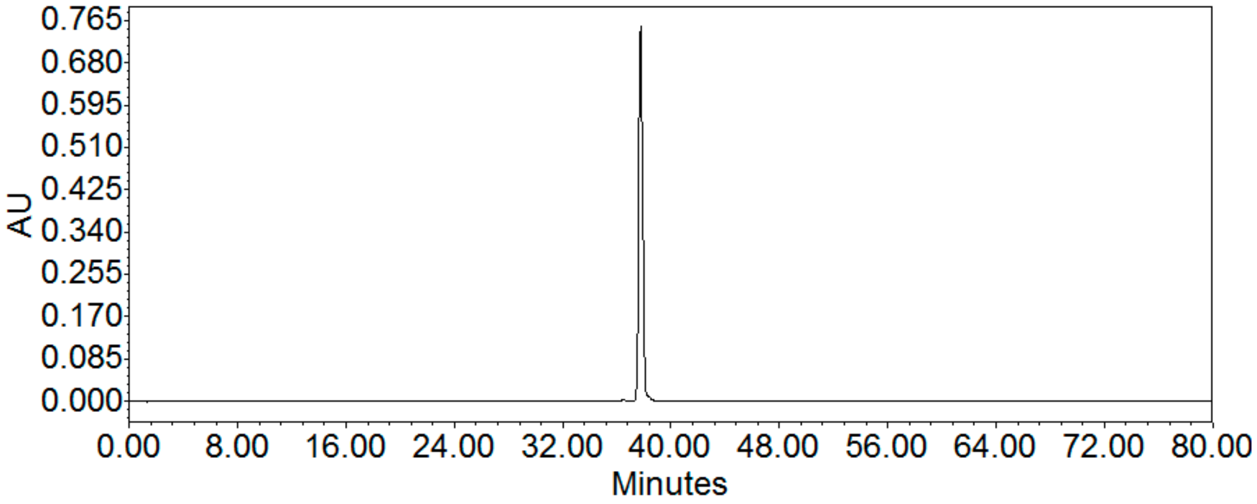

Figure S11. Chromatogram of kaempferol-3-glucoside standard

4. **Examples of chromatograms of phenolic compounds in analysed hop cultivars**

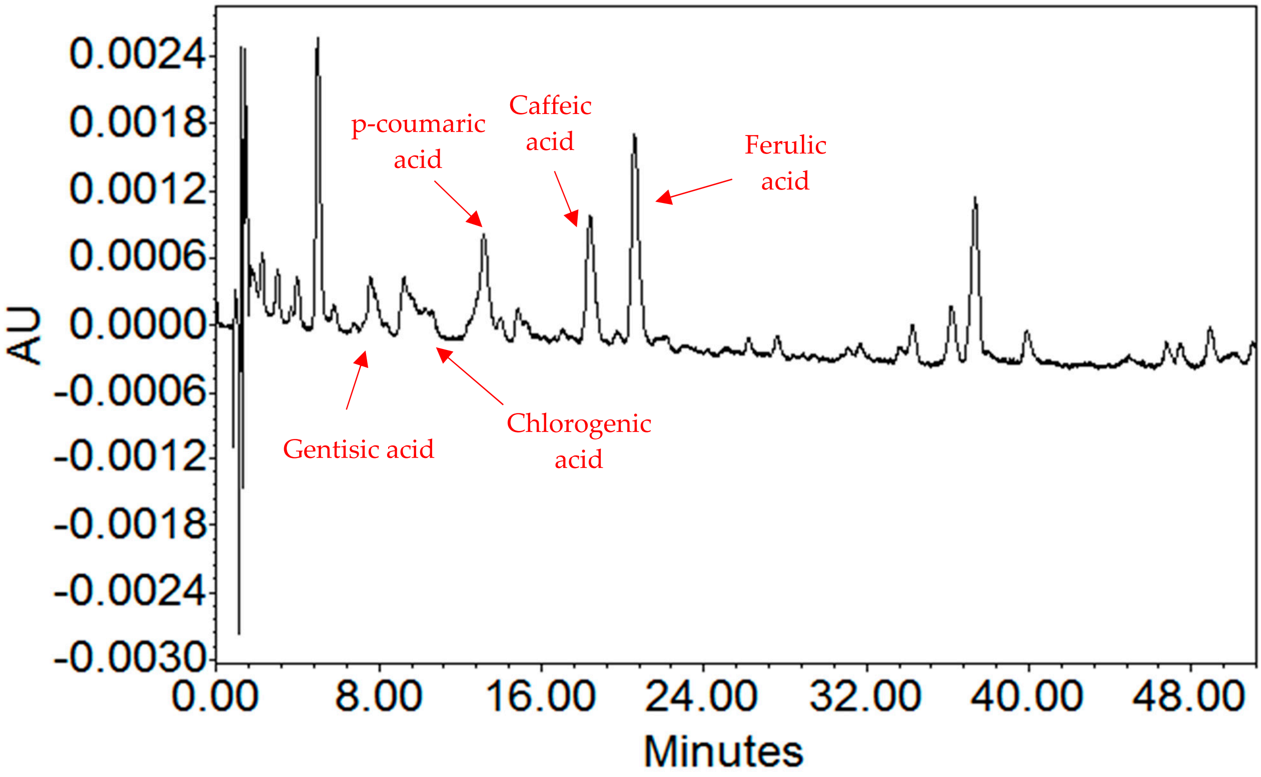

Figure S12. Chromatogram of lower hop stalks preceded by hydrolysis in Marynka cultivar at 320 nm

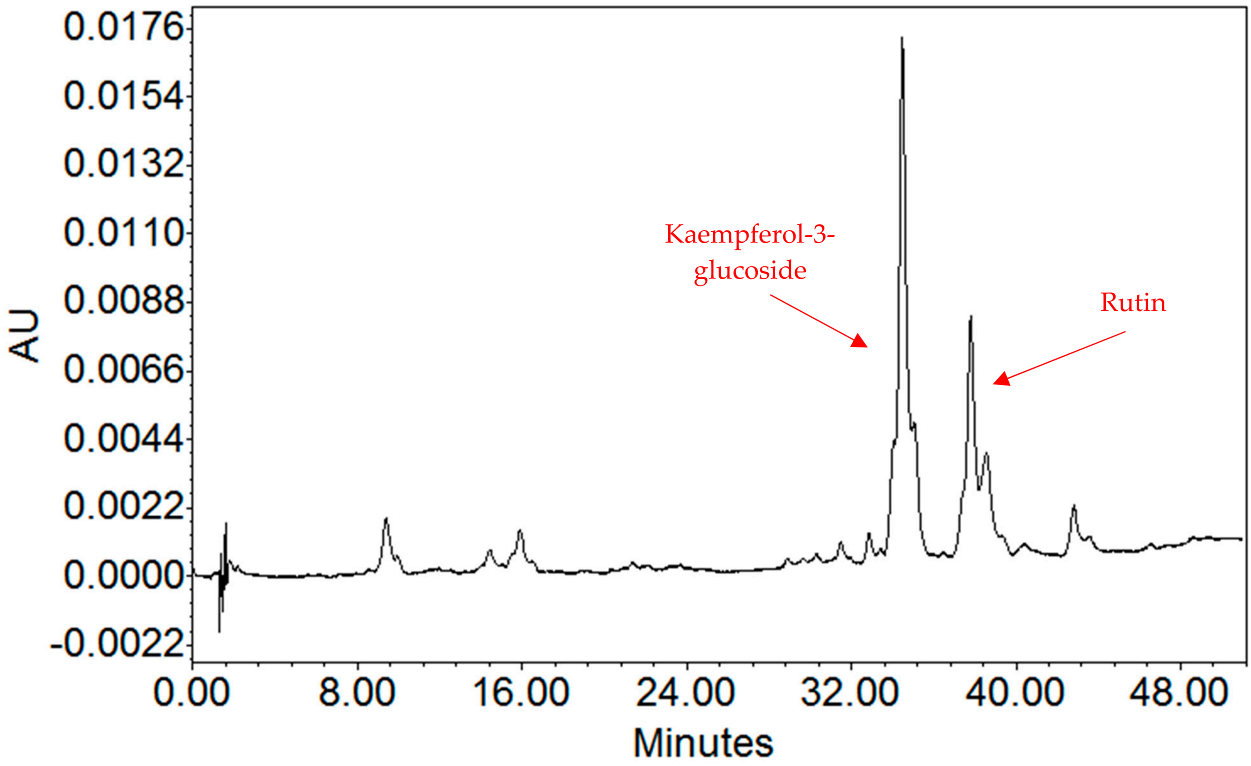

Figure S13. Chromatogram of upper hop cones in Marynka cultivar at 355 nm

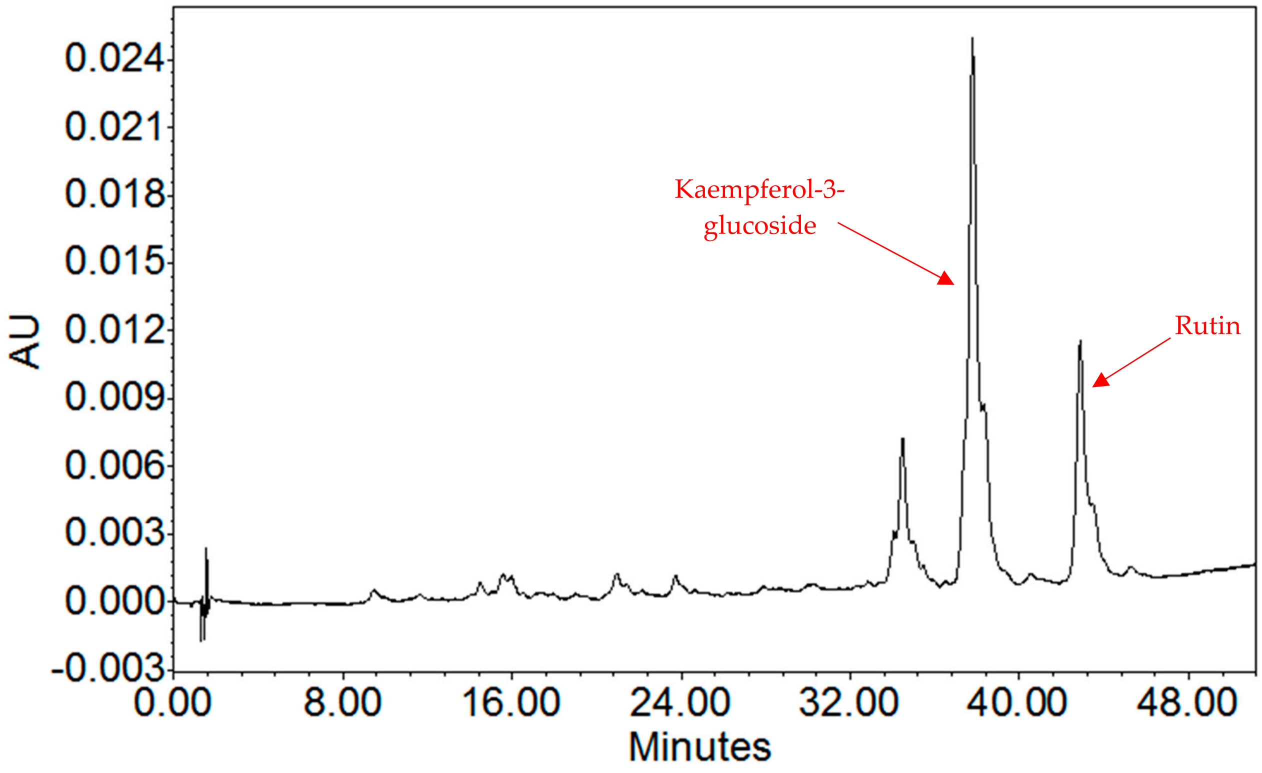

Figure S14. Chromatogram of middle hop leaves in Lubelski cultivar at 355 nm

5. Calibrations curve used to quantitative estimation of phenolic compounds in analysed hop cultivars

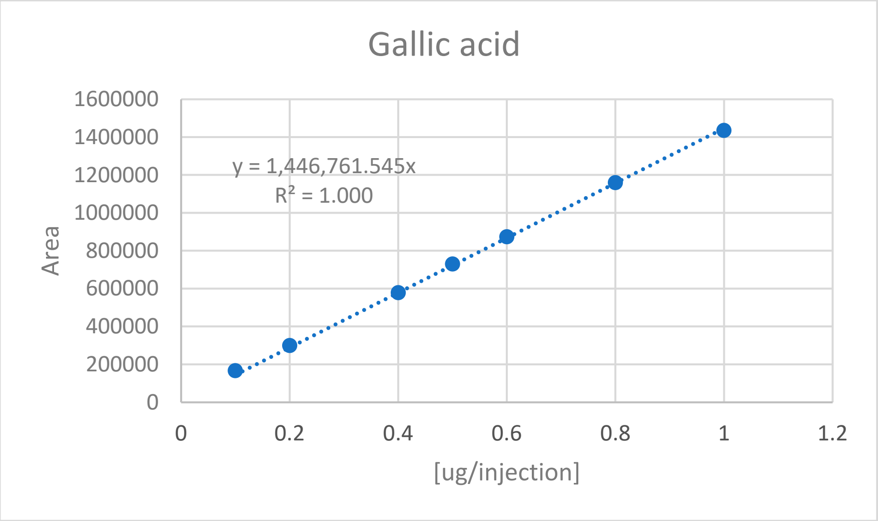

Figure S15. Calibration curve for the quantitative estimation of gallic acid

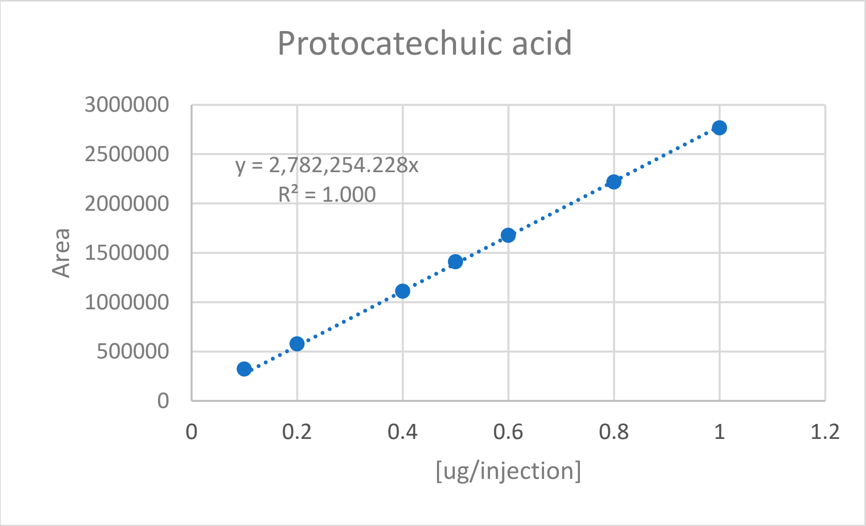

Figure S16. Calibration curve for the quantitative estimation of protocatechuic acid

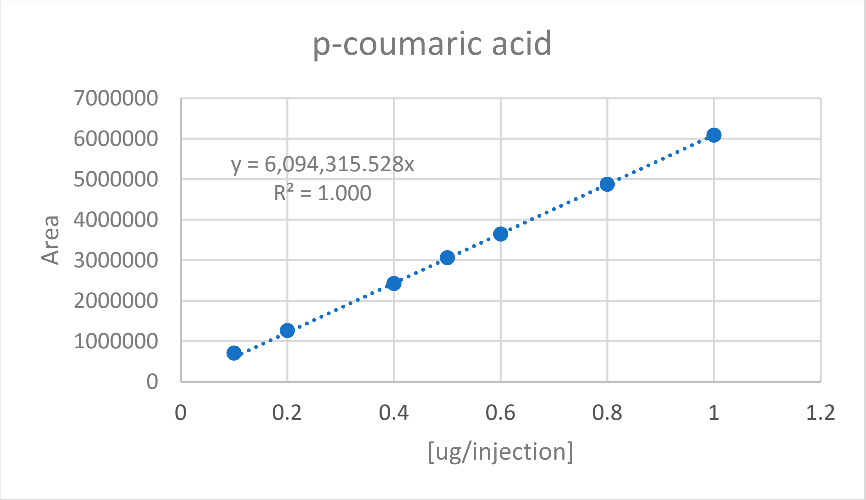

Figure S17. Calibration curve for the quantitative estimation of p-coumaric acid

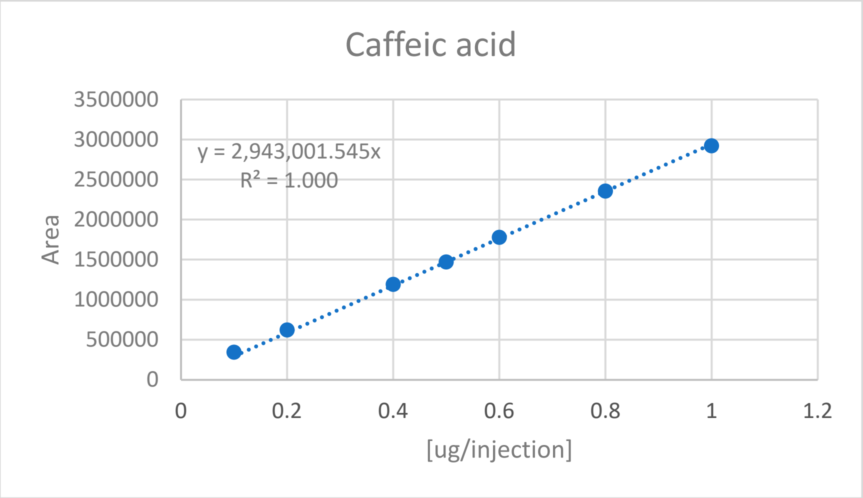

Figure S18. Calibration curve for the quantitative estimation of caffeic acid

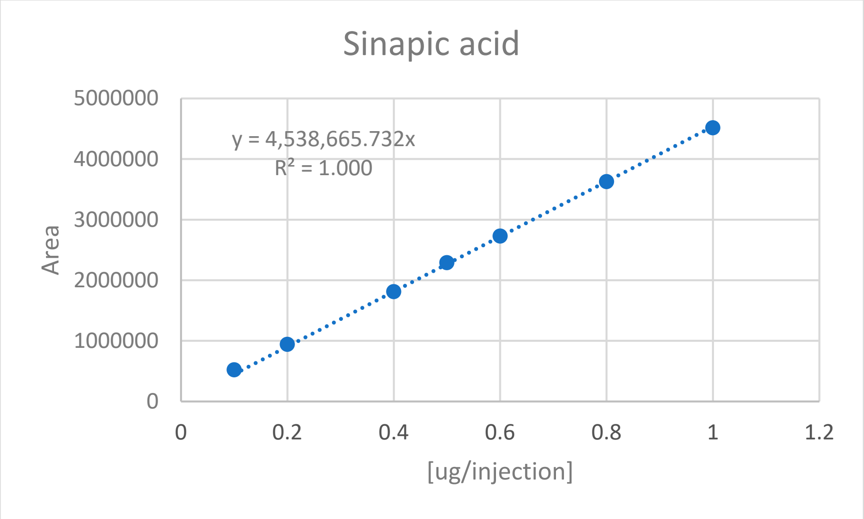

Figure S19. Calibration curve for the quantitative estimation of sinapic acid

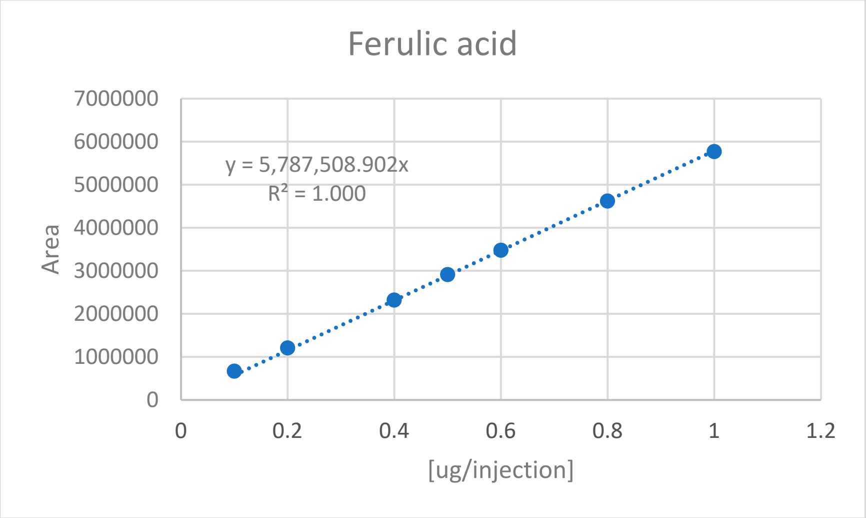

Figure S20. Calibration curve for the quantitative estimation of ferulic acid

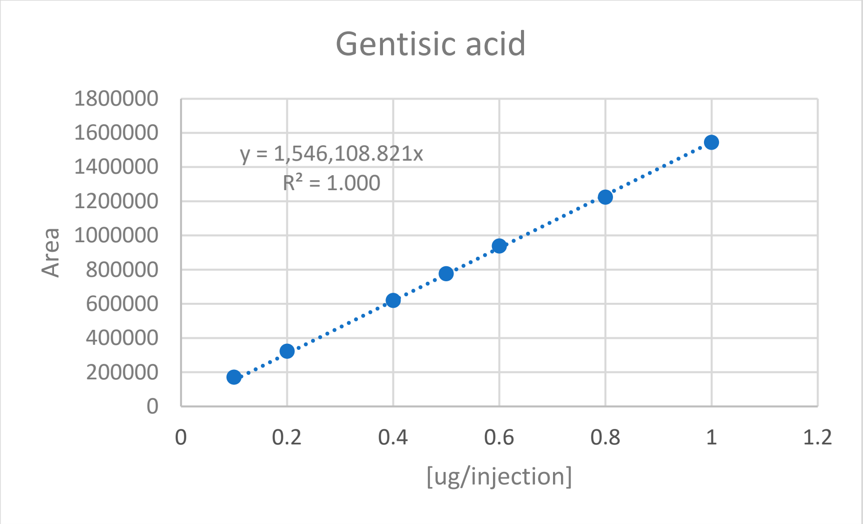

Figure S21. Calibration curve for the quantitative estimation of gentisic acid

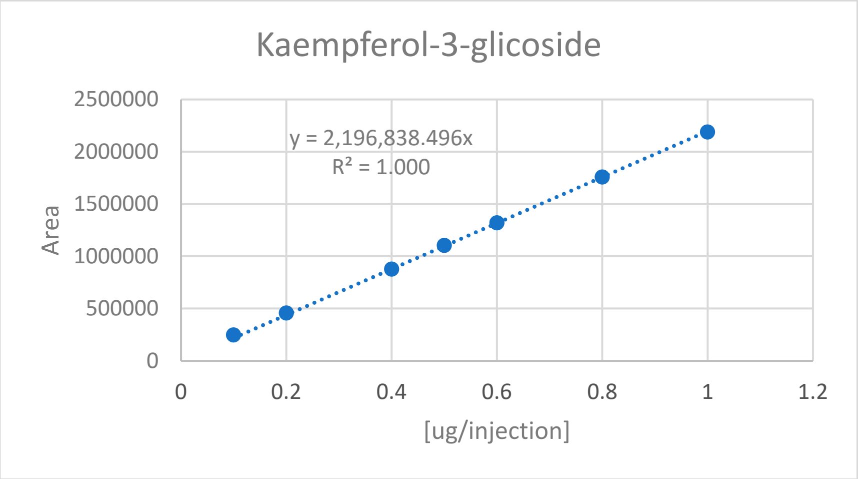

Figure S22. Calibration curve for the quantitative estimation of kaempferol-3-glucoside

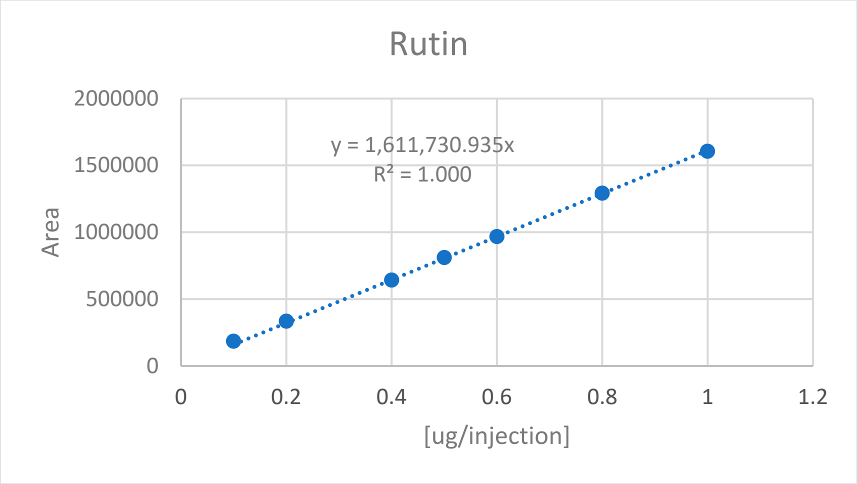

Figure S23. Calibration curve for the quantitative estimation of rutin
